# lncRNA MALAT1 regulated ATAD2 to facilitate retinoblastoma progression via miR-655-3p

**DOI:** 10.1515/med-2021-0290

**Published:** 2021-06-24

**Authors:** Yuxin Zhao, Zhaoxia Wang, Meili Gao, Xuehong Wang, Hui Feng, Yuanyuan Cui, Xia Tian

**Affiliations:** Department of Ophthalmology, Weihai Central Hospital, No. 3, Mishandongluxi, Wendeng District, Weihai, 264400, Shandong, China; Department of Pediatric, Weihai Central Hospital, Weihai, Shandong, China

**Keywords:** MALAT1, miR-655-3p, ATAD2, tumor progression, retinoblastoma

## Abstract

Long noncoding RNA (lncRNA) metastasis-associated lung adenocarcinoma transcript 1 (MALAT1) was reported as an oncogene in many tumors including retinoblastoma (RB). This research mainly focused on the functions and mechanism of MALAT1 in RB. MALAT1 was upregulated in RB tissues and cells, and it served as a competing endogenous RNA (ceRNA) and inhibited miRNA-655-3p (miR-655-3p) expression, which eventually regulated the expression of miR-655-3p downstream target ATPase Family AAA Domain Containing 2 (ATAD2). The level of ATAD2 significantly increased, while that of miR-655-3p remarkably decreased in RB tissues and cells. MALAT1 depletion inhibited cell proliferation, metastasis, and epithelial–mesenchymal transition (EMT), but promoted apoptosis *in vitro* and blocked xenograft tumor growth *in vivo*. MALAT1 exerted its oncogenic functions in RB by regulating miR-655-3p/ATAD2 axis.

## 1 Introduction

Retinoblastoma (RB), originated from the retina, is the most aggressive intraocular cancer in children, which seriously threatened the infant vision and lives [1]. With the improvement of treatment, the survival of RB patients is almost 100% [2]. To some extent, the high cure rate of RB is based on the accurate diagnosis in the early stage [3]. For example, the survival rate of RB in Africa was only 20–46% because of the diagnosis at advanced stage [4]. Thus, searching for effective treatment methods and biomarkers is still essential for RB patients, especially for patients who have suffered the spread of cancer.

Long noncoding RNAs (lncRNAs) are a category of long RNAs (>200 nucleotides [nt]) with no translation capacity and could affect target gene expression at the transcriptional stage [5]. Mounting evidence has indicated the enormous potential of lncRNAs as novel biomarkers and therapeutic targets for cancer [6]. Until now, several lncRNAs have been reported to be correlated with the progression of RB and exerted tumor promoter or inhibitor role in RB. For example, long intergenic nonprotein coding RNA 202-1 (LINC00202) was highly expressed in RB, and its silencing retarded cell growth and metastasis *in vitro* [7]. lncRNA THOR (ENSG00000226856) was upregulated in RB; the depletion of THOR resulted in the decrease in cell growth and metastasis and the increase in apoptosis [8]. Metastasis-associated lung adenocarcinoma transcript 1 (MALAT1) is a highly conserved noncoding RNA. Amounting evidence indicated that the MALAT1 was highly expressed in various types of cancer, including ovarian cancer [9], gastric cancer (GC) [10], colorectal cancer [11], papillary thyroid cancer [12], and non-small cell lung cancer [13]. A recent research verified that MALAT1 was highly expressed in RB tissues, and MALAT1 depletion inhibited the proliferation of RB cells, which indicated the pivotal role of MALAT1 in RB [14]. However, the molecular mechanism of MALAT1 in RB was inadequately explained.

MicroRNAs (miRNAs), a form of small RNAs (∼18–25 nt) without the ability of translation, were identified to influence the expression of the target gene by targeting messenger RNA (mRNA) [15]. The dysregulation of miRNAs was documented in RB. For instance, the low expression of miR-125a-5p in RB promoted cell growth [16]. Similarly, miR-3619-5p [7] and miR-758 [17] were downregulated in RB and negatively associated with the RB progression. However, miR-492 [18] was upregulated in RB tissues and its depletion could impede the malignant behavior of RB. miR-655-3p was encoded by a polycistronic miRNA gene cluster in the human chromosomal locus 14q32. This locus-encoded miRNAs participated in the regulation of adhesion, invasion, and motility pathways [19]. Not surprisingly, miR-655-3p was reported to function as a tumor suppressor, and its overexpression decreased HCC cell proliferation, migration, and invasion [20]. Besides, the effective delivery of miR-655-3p using nanoscale coordination polymers (NCPs) exhibit tumor-suppressive effects on advanced metastatic liver tumors [21]. Research also showed that miR-655-3p impacted cell behaviors of tumor progression in ovarian cancer [22], non-small cell lung cancer [23], and glioma [24]. These results indicating the enormous potential of miR-655-3p as therapeutic candidate target for cancer treatment. However, no study has been published to show the effect and mechanism of miR-655-3p in RB.

It is generally accepted that lncRNA as the competing endogenous RNA (ceRNA) regulates miRNAs, thus affecting the transfection and translation of downstream genes. ceRNAs are RNA transcripts which can function by decreasing targeting concentration of miRNA and derepressing other mRNAs containing the common miRNA response elements (MREs) [25]. Sufficient research evidence supported that this novel RNA cross talk exerted pivotal role in human health and disease. lncRNA highly upregulated in liver cancer (HULC) could reduce miR-372 expression and activity and then reduce translational repression of its target transcript gene [26]. Nuclear Enriched Abundant Transcript 1 (NEAT1) competed for miR-506 in GC and modulated the expression of signal transducer and activator of transcription 3 (STAT3), which ultimately caused an increase in growth, invasion, and migration [27]. In human RB, lncRNA X inactive-specific transcript (XIST) [28], differentiation antagonizing nonprotein coding RNA (DANCR) [29], and homeobox A11 antisense RNA (HOXA11-AS) [30] were found that functioned as ceRNAs for its target miRNA and regulate its endogenous targets and affect the progression of RB. Therefore, understanding ceRNA cross talk will expand the understanding in gene regulatory networks and provide new treatment strategies and methods for RB.

In this research, we first detected the expression of MALAT1 and miR-655-3p in RB. The function and the correlation between MALAT1 and miR-655-3p were explored. Besides, we also detected the downstream target of MALAT1/miR-655-3p axis in RB. Moreover, a mouse xenograft model was employed to analyze the effect of MALAT1 knockdown on RB *in vivo*. These findings indicate the possibility of MALAT1 as a diagnostic biomarker or a therapeutic target for RB.

## 2 Materials and methods

### 2.1 Tissue collection

Thirty RB tissue samples and 18 matched normal globe tissue samples were collected from Weihai Central Hospital. Normal globe tissues were from patients who had suffered ruptured globes. All patients were not subjected to radiation therapy and chemotherapy before enucleation. All tissues were frozen at −80°C immediately after resection until use. The research was permitted by the Ethics Committee of Weihai Central Hospital and carried out according to the Declaration of Helsinki Principles. Written informed consents were provided by all patients.

### 2.2 Cell culture and transfection

Human RB cell lines (Y79 and WERI-Rb-1) and adult retinal pigment epithelial cell line ARPE19 were purchased from American Type Culture Collection (ATCC). All cells were cultivated with RPMI-1640 medium (Biosun, Shanghai, China) containing 10% fetal bovine serum (FBS; Biosun) in 5% CO_2_ incubator at 37°C. Small interfering RNA (siRNA) targeting MALAT1 (si-MALAT1, 5′-CACAGGGAAAGCGAGTGGTTGGTAA-3′) and its negative control (si-con, 5′-UUCUCCGAACGUGUCACGUTT-3′), miR-655-3p mimics (miR-655-3p, 5′-AUAAUACAUGGUUAACCUCUUU-3′) and its negative control (miR-con, 5′- UUCUCCGAACGUGUCACGUTT-3′), miR-655-3p inhibitor (anti-miR-655-3p, 5′-AAAGAGGUUAACCAUGUAUUAU-3′) and its negative control (anti-miR-con, 5′-UUGUACUACACAAAAGUACUG-3′), and ATAD2 overexpression vector (pcDNA-ATAD2) and empty vector (pcDNA) were all obtained from Sangon Biotech (Shanghai, China). The increased or decreased expression of miR-655-3p was achieved by transfecting miR-655-3p mimics or miR-655-3p inhibitor. Transfection of si-MALAT1 induced the decreased expression of MALAT1, while ATAD2 overexpression was achieved by transfecting pcDNA-ATAD2. Transfection was conducted using Lipofectamine 2000 (Invitrogen, Carlsbad, CA, USA) according to the manufacturer’s instructions. Each group of cells was harvested for 24 or 48 h after transfection for further assays.

### 2.3 Quantitative real time reverse transcription polymerase chain reaction (RT-qPCR)

Total RNA was extracted from RB tissue samples or tumor cells using TRIzol (Thermo Fisher Scientific, USA) reagent according to the manufacturer’s instructions. To determine the expression of miR-655-3p, RT-qPCR was performed using a Mir-X™ miRNA RT-qPCR TB Green® Kit (638314; TaKaRa, Japan) on a real time detection system (Bio-Rad, Shanghai, China). To analyze the expression of MALAT1 and ATAD2, a PrimeScript™ RT reagent Kit (RR037A; TaKaRa) was used to synthesize the cDNA and TB Green® Premix Ex Taq™ II (RR820A; TaKaRa) was used to detect the expression levels of MALAT1 and ATAD2. The levels of MALAT1 and ATAD2 were normalized by glyceraldehyde 3-phosphate dehydrogenase (GAPDH), while the level of miR-655-3p was normalized by small nuclear RNA U6 and processed by the 2^−ΔΔCt^ method. The primers were obtained from Songon (Shanghai, China) and presented as follows: MALAT1 (F: 5′-AATGTTAAGAGAAGCCCAGGG-3′, R: 5′-AAGGTCAAGAGAAGTGTCAGC-3′), miR-655-3p (F: 5′-CGCGCGATAATACATGGTTAAC-3′, R: 5′-GTGTCTTAAGGCTAGGCCTA-3′), ATAD2 (F: 5′-GGAATCCCAAACCACTGGACA-3′, R: 5′-GGTAGCGTCGTCGTAAAGCACA-3′), GAPDH (F: 5′-GGAAATGAATGGGCAGCCGT-3′, R: 5′-GTTAAAAGCAGCCCTGGTGAC-3′), and U6 (F: 5′-CTCGCTTCGGCAGCACA-3′, R: 5′-AACGCTTCACGAATTTGCGT-3′).

### 2.4 Cell counting kit-8 (CCK-8) assay

A CCK-8 kit (Beyotime, Shanghai, China) was utilized to assess cell viability. The Y79 and WERI-Rb-1 cells (2 × 10^4^ per well) were seeded into the 96-well plate and maintained for 24 h. At the end of the incubation time, CCK-8 solution was added into each well at 0, 24, 48, and 72 h, and the optical density at 450 nm was assessed by a Multiscan Spectrum (Tecan, Switzerland).

### 2.5 Flow cytometry analysis of cell apoptosis

An Annexin V-FITC/PI Apoptosis Detection Kit (Vazyme, Nanjing, China) was used to detect the apoptosis rate. In brief, the transfected Y79 and WERI-Rb-1 cells were collected and washed with phosphate buffer solution (PBS) three times. Then cells were incubated with Annexin V–fluorescein isothiocyanate (FITC) and propidium iodide (PI) at 4°C for 15 min in dark condition. Cells were analyzed by flow cytometry (Agilent, Beijing, China).

### 2.6 Western blot assay

Total protein in Y79 and WERI-Rb-1 cells was extracted using a RIPA Lysis buffer (Solarbio, Beijing, China) supplemented with phenylmethanesulfonyl fluoride (1 mM, Beyotime, China), and the concentration of protein samples was detected by a BCA Protein Assay Kit (Beyotime). Equal amount of protein samples were separated by sodium dodecyl sulfonate-polyacrylamide gel electrophoresis and then transferred onto the polyvinylidene fluoride membrane (Millipore, Billerica, MA, USA). The membrane was blocked with 5% skim milk for 1 h and incubated with primary antibody for 12 h at 4°C. Then the membrane was incubated with secondary antibody for 2 h at room temperature. The chemiluminescence intensity was detected using an ECL kit (Beyotime) at a BIO-RAD imaging system (Bio-Rad, CA, USA). The primary antibodies p21 (1/1,500; ab218311), CyclinD1 (1/1,000; ab40754), B-cell lymphoma-2 (Bcl-2; 1/1,000; ab32124), cleaved caspase 3 (cleaved-casp-3, 1/500; ab32042), E-cadherin (ab40772, 1/2,000), N-cadherin (ab18203, 1/2,000), Vimentin (ab45939, 1/1,000), ATPase family AAA domain containing 2 (ATAD2, 1/500; ab176319), GAPDH (1/2,500; ab9485), and secondary antibody Goat Anti-Rabbit IgG H&L (HRP) (1/10,000; ab97051) were purchased from Abcam (Cambridge, MA, USA).

### 2.7 Transwell assay

Transwell chambers (Solarbio) were utilized to monitor the migrated and invaded abilities of Y79 and WERI-Rb-1 cells. For the migration assay, RPMI-1640 medium containing 10% FBS was supplemented into the lower chamber, while the upper chamber was added with suspended cells in serum-free medium. After 24-h incubation, the migrated cells were stained with 0.1% crystal violet. Cell numbers in ten random fields were counted using a light microscope and calculated using Image Pro Plus. The protocol of invasion assay was similar to that of the migration assay, while the difference was that the upper chamber was covered with a matrigel matrix (BD, Franklin Lakes, USA).

### 2.8 Dual-luciferase reporter assay

The wide-type and mutant complementary sequences of MALAT1 or 3′-untranslated regions (3′-UTR) of ATAD2 were inserted into pGL3 vector (Promega, Madison, WI, USA) to construct the luciferase reporter WT-MALAT1, MUT-MALAT1, WT-ATAD2, or MUT-ATAD2. Y79 and WERI-Rb-1 cells were co-transfected with luciferase reporter and miR-655-3p or miR-con. The cells were harvested after 48 h and the luciferase activity was evaluated by using a Dual-Lucy Assay Kit (Solarbio). Renilla luciferase values were used to normalize the Firefly luciferase values.

### 2.9 Mouse xenograft experiments

Y79 cells stable expressing sh-MALAT1 or sh-con were produced by inserting sh-MALAT1 or sh-con into the lentiviral vector pLKO.1-puro lentiviral vectors (Sigma, Merck KGaA, Darmstadt, Germany). Then the cells were selected 24 h after transfection with 1 µg/mL puromycin (Sigma) for 10–14 days.

For the RB tumor xenograft mouse model construction, 4-week-old nude mice that were bought from Shanghai Laboratory Animal Company (SLAC, Shanghai, China) was used. 4 × 10^6^ stable MALAT1 knockdown Y79 cells with sh-MALAT1 or sh-con were injected into the right lateral flanks of the nude mice. The mice were divided into two groups (*n* = 6 per group): the sh-MALAT1 group and the sh-con group. The tumor volume was measured every 7 days to 28 days and tumor size was calculated according to the formula: volume = width^2^ × length/2. After 28 days, the mouse was executed and xenograft tumors were resected and weighed. The tumor tissues were stored in a −80°C refrigerator for further exploration. All animal experiment procedures were approved by the Animal Care Committee of Weihai Central Hospital.

### 2.10 Statistical analysis

GraphPad Prism 7 (GraphPad Inc., La Jolla, CA, USA) was used to process the experimental data which were performed in triplicate. The unpaired Student’s *t*-test was used to analyze the difference between the two groups, and one-way analysis of variance (ANOVA) was utilized to analyze the differences among multiple groups. Pearson correlation analysis was used to test the association among the expression of MALAT1, miR-655-3p, and ATAD2. *P* < 0.05 was considered statistically significant.

## 3 Results

### 3.1 MALAT1 was upregulated, while miR-655-3p was downregulated in RB tissues

**Figure 1:**
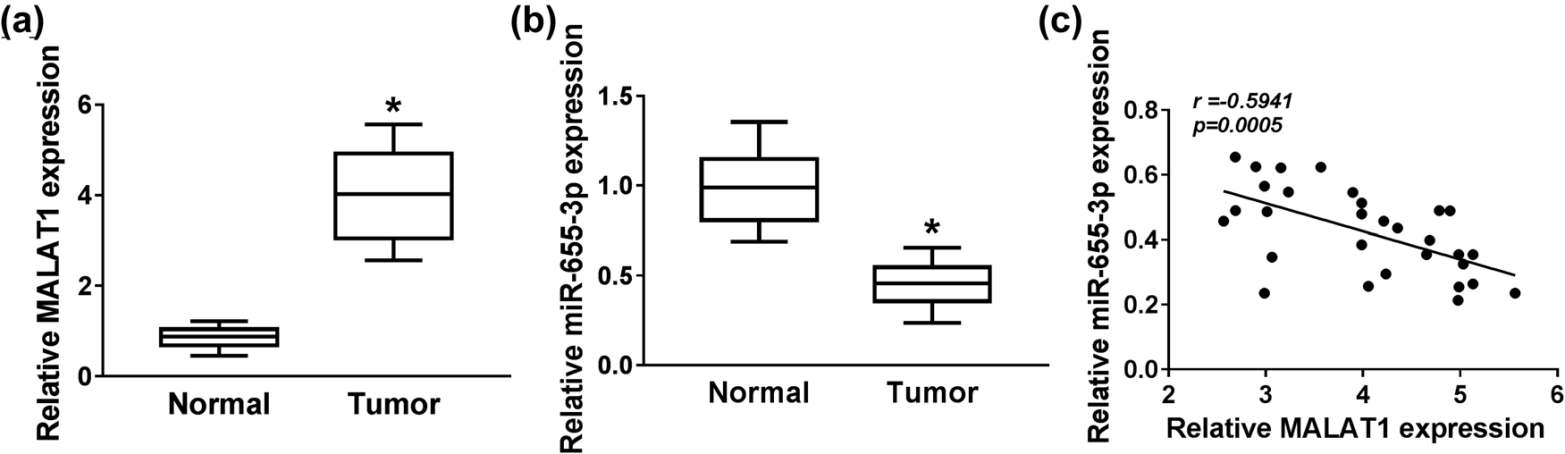
MALAT1 was upregulated, while miR-655-3p was downregulated in RB tumor tissues. (a and b) The expression of MALAT1 and miR-655-3p in RB tumor tissues and normal globe tissues were tested by RT-qPCR. (c) The correlation between miR-655-3p and MALAT1 was verified by Pearson’s correlation analysis. *P < 0.05.

The expressions of long noncoding RNA MALAT1 and miRNA-655-3p in 30 RB tissue samples and 18 normal globe tissue samples were detected first. As displayed in Figure 1a and b, the expression of MALAT1 was apparently elevated in RB tissues compared to that in normal tissues. Conversely, the expression of miR-655-3p was distinctly decreased in RB tissues (Figure 1b). Besides, Pearson’s correlation analysis exhibited that the expression of miR-655-3p in RB was negatively correlated with MALAT1 expression (Figure 1c). These data indicated that MALAT1 was highly expressed and miR-655-3p was lowly expressed in RB tissues.

### 3.2 MALAT1 silencing constrained cell proliferation, metastasis, and EMT but promoted apoptosis in Y79 and WERI-Rb-1 cells

**Figure 2:**
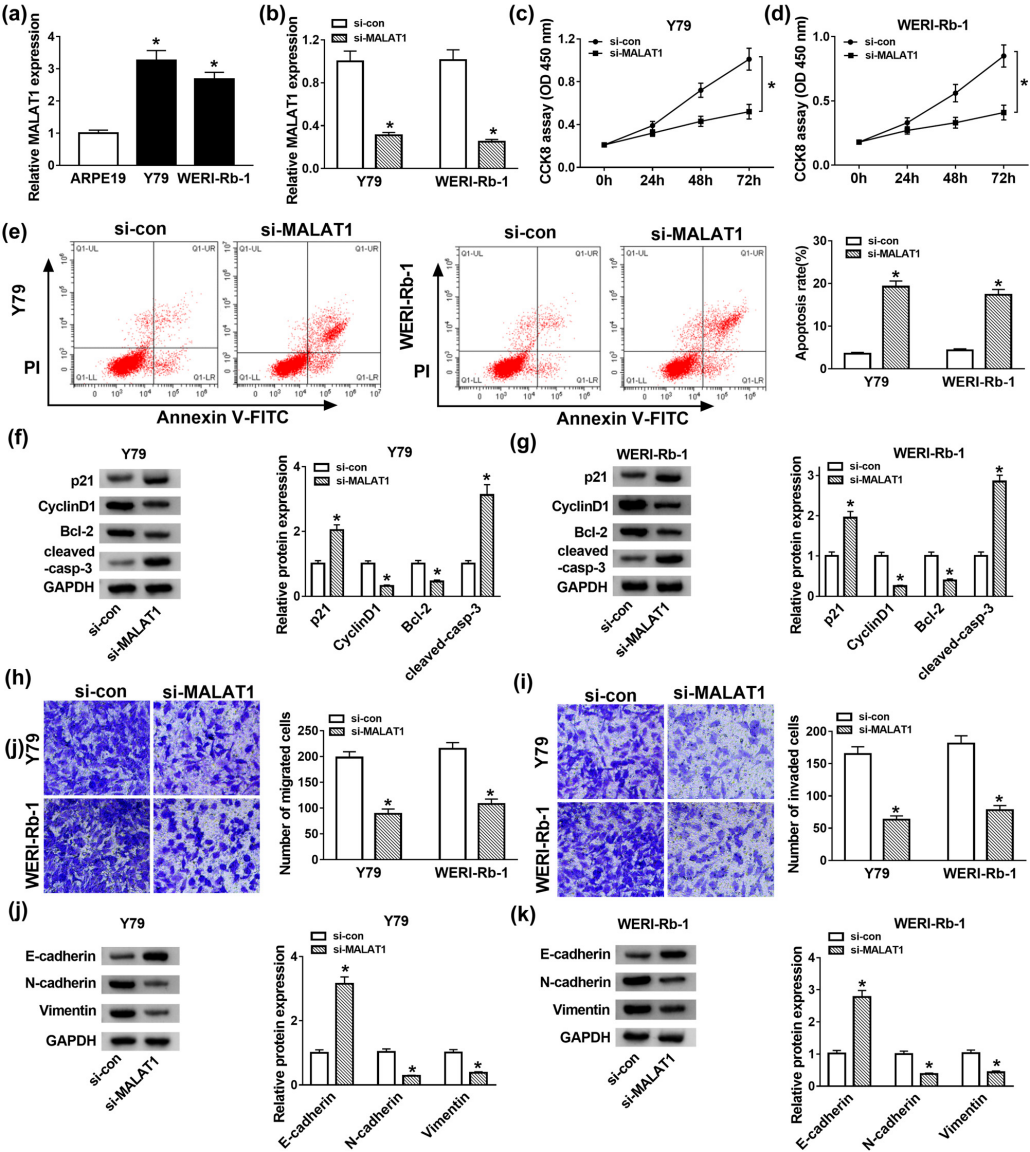
MALAT1 depletion suppressed cell proliferation but induced apoptosis and EMT in Y79 and WERI-Rb-1 cells. (a) The expression of MALAT1 in RB cells (Y79 and WERI-Rb-1) and retinal pigment epithelial cell ARPE19 was detected by RT-qPCR. (b–k) Y79 and WERI-Rb-1 cells were transfected with si-NC or si-MALAT1. (b) The expression of MALAT1 in Y79 and WERI-Rb-1 cells was examined by RT-qPCR after transfection for 24 h. (c and d) The cell viability was detected by CCK-8 assay after transfection for 0, 24, 48, and 72 h. (e) The apoptosis rate was assessed by flow cytometry after transfection for 48 h. (f and g) The protein levels of p21, Cyclin D1, Bcl-2, and cleaved-casp-3 were detected by western blot after transfection for 48 h. (h and i) After transfection for 24 h, cell migration and invasion were detected by transwell assay. (j and k) The protein levels of E-cadherin, N-cadherin, and Vimentin were examined by western blot after transfection for 48 h. *P < 0.05.

Consistent with its expression in RB tissues, MALAT1 expression in RB cell lines (Y79 and WERI-Rb-1) was obviously increased in contrast with the retinal pigment epithelial cell line ARPE19 (Figure 2a). To investigate the functions of MALAT1 in RB, Y79 and WERI-Rb-1 cells were transfected with si-NC or si-MALAT1. As exhibited in Figure 2b, transfection of si-MALAT1 successfully reduced the expression of MALAT1 compared with the si-NC group. Moreover, the transfection of si-MALAT1 resulted in a marked decline of cell viability in Y79 and WERI-Rb-1 cells in comparison with the si-con group (Figure 2c and d). Flow cytometry assay showed that the apoptosis rate of cells with MALAT1 depletion was notably facilitated in Y79 and WERI-Rb-1 cells transfected with si-MALAT1 (Figure 2e).

Cyclin D1 phosphorylates the RB protein and drives G1 to S phase progression by forming active complexes with cyclin-dependent kinases (CDKs), and its abnormal elevated expression in tumors promoted cell growth and ultimately accelerated neoplastic growth [31]. p21, a member of Cip/Kip family of CKIs, exerted proliferation inhibitor and apoptosis inhibitor roles in cancers [32]. Since p21 and Cyclin D1 reflect the cell proliferation and Bcl-2 (anti-apoptotic protein) and cleaved-casp-3 were apoptosis-associated proteins, we further confirmed their protein levels in Y79 and WERI-Rb-1 cells. The western blot assay exhibited that the protein levels of p21 and cleaved-casp-3 were strikingly enhanced, but the protein levels of Cyclin D1 and Bcl-2 were conspicuously decreased in si-MALAT1-transfected Y79 and WERI-Rb-1 cells (Figure 2f and g). Besides, the introduction of si-MALAT1 notably reduced the migration and invasion abilities of Y79 and WERI-Rb-1 cells compared to the si-con group (Figure 2h and i). Epithelial–mesenchymal transition (EMT) was the most common primary reason for tumor invasion and metastasis [33]. The hallmark of EMT is the upregulation of epithelial markers including N-cadherin and Vimentin followed by the downregulation of E-cadherin [34]; thus, we further detected the levels of these proteins. As exhibited in Figure 2j and k, the protein levels of E-cadherin were apparently elevated, while N-cadherin and Vimentin were strikingly decreased in the si-MALAT1 group in comparison with that in the si-con group. Taken together, the depletion of MALAT1 inhibited cell proliferation, migration, invasion, and EMT but promoted apoptosis in Y79 and WERI-Rb-1 cells.

### 3.3 MALAT1 negatively interacted with miR-655-3p in Y79 and WERI-Rb-1 cells

**Figure 3:**
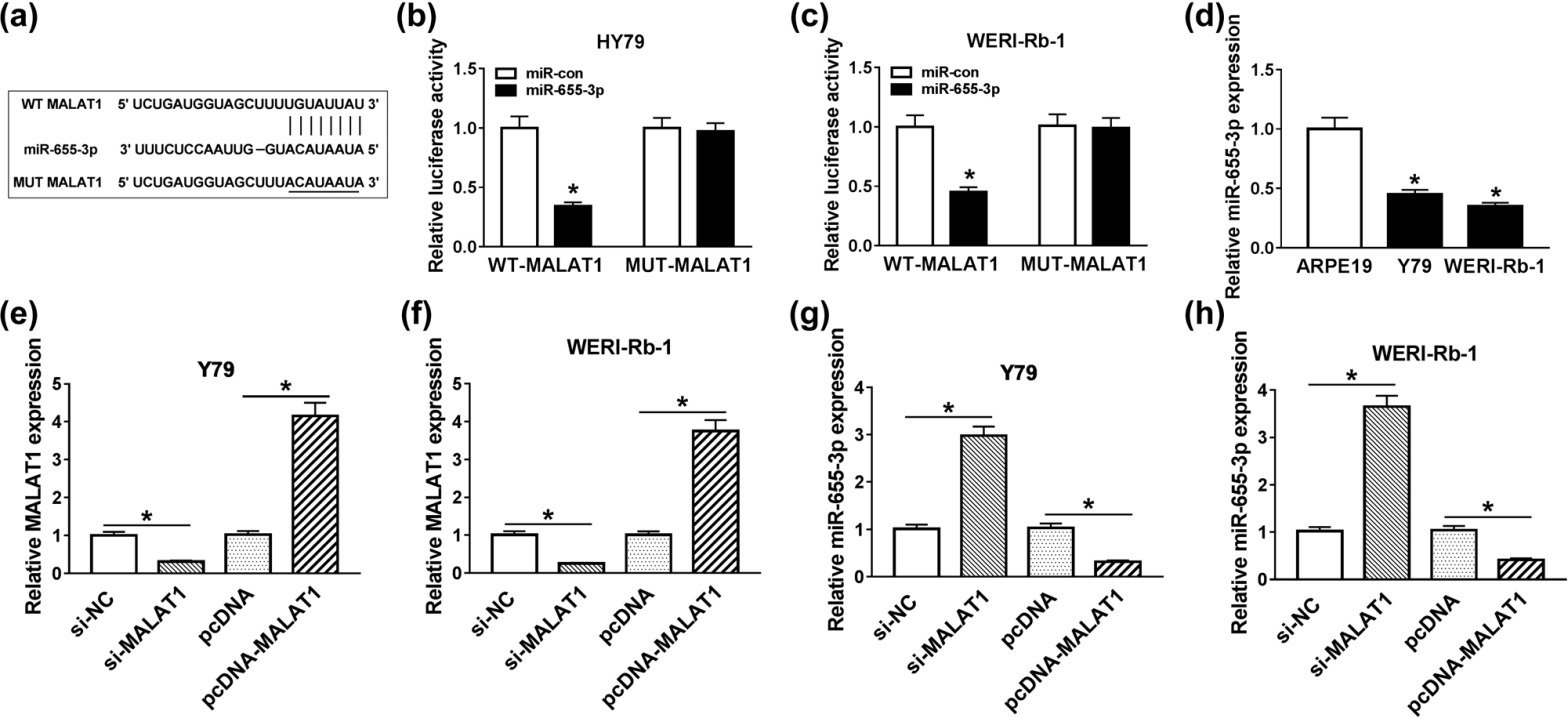
MALAT1 negatively interacted with miR-655-3p in Y79 and WERI-Rb-1 cells. (a) The fragment of MALAT1 containing the putative or mutant miR-655-3p binding sites. (b and c) WT-MALAT1 or MUT-MALAT1 reporter plasmids and miR-con or miR-655-3p were co-transfected into Y79 and WERI-Rb-1 cells for 48 h. The luciferase activities were evaluated by dual-luciferase reporter assay. (d) The expression of miR-655-3p in RB cells Y79 and WERI-Rb-1 and retinal pigment epithelial cell line ARPE19 was tested by RT-qPCR. (e–h) Y79 and WERI-Rb-1 cells were transfected with si-NC, si-MALAT1, pcDNA, or pcDNA-MALAT1 for 24 h. (e and f) The expression of MALAT1 in the cells upon transfection was detected by RT-qPCR. (g and h) The expression of miR-655-3p in Y79 and WERI-Rb-1 cells upon transfection was detected via RT-qPCR. *P < 0.05.

As is widely known, lncRNAs could function as a ceRNA to specific miRNAs and decrease miRNA expression. We wondered if miR-655-3p was a target miRNA of MALAT1. StarBase online database (http://starbase.sysu.edu.cn/starbase2/) was used to predict the putative targets of MALAT1. The results showed that miR-655-3p contains the complete binding sites in MALAT1 (Figure 3a). Subsequent dual-luciferase reporter assay confirmed this interaction, as transfection of miR-655-3p induced a remarkable reduction of luciferase activity in the WT-MALAT1 group in contrast with the miR-con group, while it has little change in the luciferase activity of MUT-MALAT1 group (Figure 3b and c). Besides, the expression of miR-655-3p was evidently downregulated in Y79 and WERI-Rb-1 cells compared with the ARPE19 cells (Figure 3d). Moreover, Y79 and WERI-Rb-1 cells were transfected with si-NC, si-MALAT1, pcDNA, or pcDNA-MALAT1, and the expressions of MALAT1 and miR-655-3p were detected. As shown in Figure 3e–h, transfection of si-MALAT1 inhibited the expression of MALAT1 but suppressed miR-655-3p expression. Instead, MALAT1 expression was elevated and miR-655-3p was downregulated when cells were transfected with pcDNA-MALAT1. These data implicated that MALAT1 suppressed the expression of miR-655-3p by binding directly to the latter.

### 3.4 MALAT1 knockdown inhibited cell proliferation but facilitated apoptosis in RB by sponging miR-655-3p

**Figure 4:**
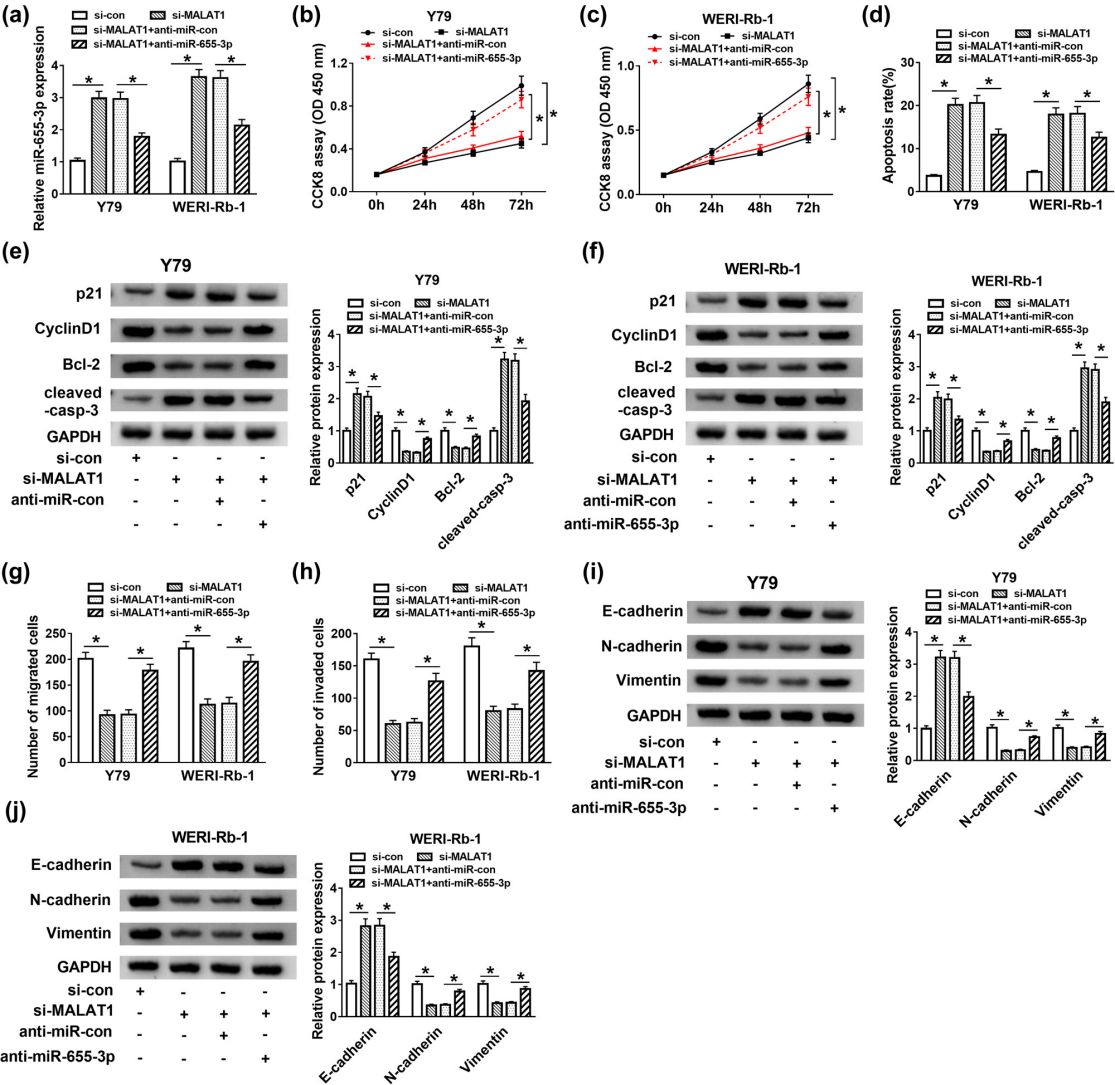
MALAT1 regulated RB progression by sponging miR-655-3p. (a–j) The Y79 and WERI-Rb-1 cells were transfected with si-con, si-MALAT1, si-MALAT1 + anti-miR-con, or si-MALAT1 + anti-miR-655-3p. (a) The level of miR-655-3p was measured by RT-qPCR after transfection for 24 h. (b and c) The cell viability was examined by CCK8 assay after transfection for 0, 24, 48, and 72 h. (d) The apoptosis rate was evaluated through flow cytometry after transfection for 48 h. (e and f) The protein levels of p21, Cyclin D1, Bcl-2, and cleaved-casp-3 were tested via western blot assay after transfection for 48 h. (g and h) The migration and invasion abilities were estimated by transwell assay after transfection for 24 h. (i and j) After transfection for 48 h, the protein levels of E-cadherin, N-cadherin, and Vimentin were detected by western blot. *P < 0.05.

To investigate if miR-655-3p participates in the effect of the functions of MALAT1 on RB, a loss-of-function experiment was performed. Si-con, si-MALAT1, si-MALAT1 + anti-miR-con, and si-MALAT1 + anti-miR-655-3p were transfected into Y79 and WERI-Rb-1 cells. As exhibited in Figure 4a, the expression of miR-655-3p was increased in si-MALAT1-transfected cells, but it was declined by introduction of anti-miR-655-3p. Moreover, the inhibitory effects of si-MALAT1 on cell proliferation (Figure 4b and c) and migration and invasion abilities (Figure 4g and h) of Y79 and WERI-Rb-1 cells were reversed by anti-miR-655-3p. miR-655-3p inhibitor also attenuated the promotion impact on apoptosis rate in Y79 and WERI-Rb-1 cells induced by MALAT1 silencing (Figure 4d). In addition, miR-655-3p inhibitor counteracted the elevated protein levels of p21, cleaved-casp-3, and E-cadherin as well as the declined protein levels of Cyclin D1, Bcl-2, N-cadherin, and Vimentin in Y79 and WERI-Rb-1 cells induced by si-MALAT1 (Figure 4e, f, i, and j). These data implicated that the silencing of MALAT1 suppressed RB progression by sponging miR-655-3p.

### 3.5 ATAD2 was a candidate target of miR-655-3p in Y79 and WERI-Rb-1 cells

**Figure 5:**
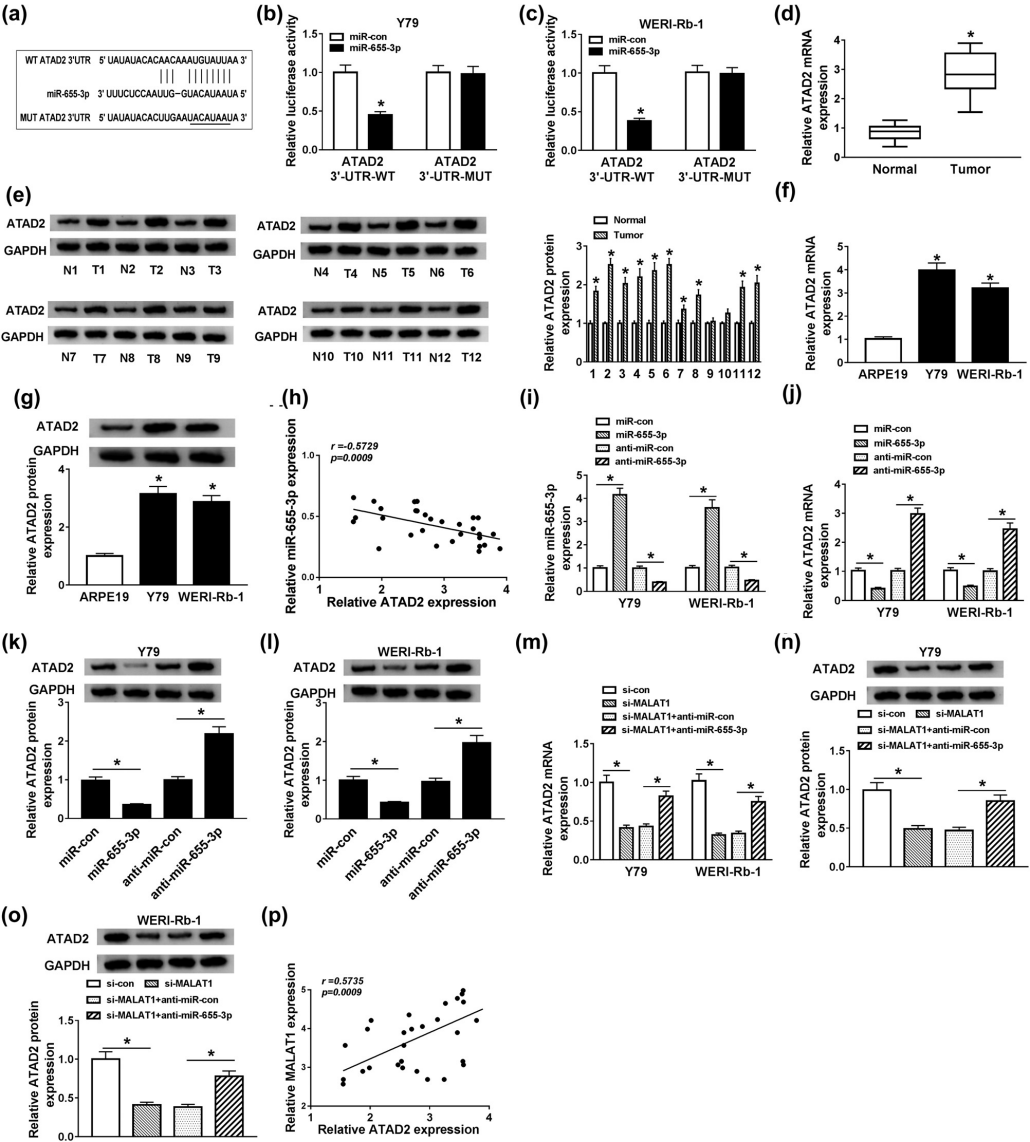
MALAT1 upregulated ATAD2 expression by repressing miR-655-3p in Y79 and WERI-Rb-1 cells. (a) Putative or mutant binding sites of miR-655-3p in the ATAD2 3′-UTR were predicted by StarBase. (b and c) The luciferase activity of Y79 and WERI-Rb-1 cells co-transfected with WT-ATAD2 or MUT-ATAD2 and miR-655-3p or miR-con for 48 h was evaluated by dual-luciferase reporter assay. (d) The mRNA level of ATAD2 in RB and normal tissues was detected by RT-qPCR. (e) The protein levels of ATAD2 in 12 RB and normal tissues were exposed by western blot assay. (f and g) The mRNA and protein levels of ATAD2 in RB cells (Y79 and WERI-Rb-1) and retinal pigment epithelial cells ARPE19 were uncovered by RT-qPCR and western blot assay, respectively. (h) The correlation between miR-655-3p and ATAD2 was processed by Pearson’s correlation analysis. (i–l) Y79 and WERI-Rb-1 cells were co-transfected with miR-con, miR-655-3p, anti-miR-con, and anti-miR-655-3p. (i and j) After transfection for 24 h, the expressions of miR-665-3p and ATAD2 mRNA were detected by RT-qPCR. (k and l) After transfection for 48 h, the protein level of ATAD2 was examined by western blot. (m–o) Y79 and WERI-Rb-1 cells were transfected with si-con, si-MALAT1, si-MALAT1 + anti-miR-con, or si-MALAT1 + anti-miR-655-3p. After transfection for 24 or 48 h, the mRNA and protein levels of ATAD2 were measured via RT-qPCR and western blot assay. (p) The correlation between MALAT and ATAD2 was validated by Pearson’s correlation analysis. *P < 0.05.

To explore the underlying mechanism of miR-655-3p in RB, starBase online database was used to search the putative target of miR-655-3p in RB. As shown in Figure 5a, ATAD2 3′-UTR had complementary binding sites with miR-655-3p. This was further confirmed by dual-luciferase assay. The luciferase activity of WT-ATAD2 transfected cells was markedly declined by transfection of miR-655-3p, while the luciferase activity of MUT-ATAD2 group had changed little when transfected with miR-655-3p (Figure 5b and c). Subsequently, ATAD2 expression in RB was uncovered. As displayed in Figure 5d–g, the mRNA and protein levels of ATAD2 in RB tissues and cells were higher than that in normal tissues and cells. Besides, a negative correlation was discovered between ATAD2 and miR-655-3p expression (Figure 5h). To investigate their relationship thoroughly, the expression of ATAD2 in cells transfected with miR-655-3p mimic or anti-miR-655-3p was detected. Transfection of miR-655-3p mimic promoted the expression of miR-655-3p (Figure 5i) but inhibited ATAD2 mRNA and protein levels (Figure 5j–l). Conversely, anti-miR-655-3p inhibited miR-655-3p expression, while the mRNA and protein levels of ATAD2 were promoted in Y79 and WERI-Rb-1 cells (Figure 5i–l). These results indicate that ATAD2 was a target of miR-655-3p and it was inhibited by miR-655-3p.

To further explore the relationship among MALAT1, miR-655-3p, and ATAD2 in RB, we detected ATAD2 expression in Y79 and WERI-Rb-1 cells transfected with si-con, si-MALAT1, si-MALAT1 + anti-miR-con, or si-MALAT1 + anti-miR-655-3p. As presented in Figure 5m–o, the levels of ATAD2 mRNA and protein were apparently downregulated in Y79 and WERI-Rb-1 cells transfected with si-MALAT1, whereas the levels were altered by introduction of anti-miR-655-3p. Besides, the expression of MALAT1 was positively correlated with ATAD2 (Figure 5p). These data demonstrated that MALAT1 could regulate ATAD2 expression by targeting miR-665-3p in Y79 and WERI-Rb-1 cells.

### 3.6 miR-655-3p negatively regulated ATAD2 expression to impede cell proliferation and impel apoptosis in Y79 and WERI-Rb-1 cells

**Figure 6:**
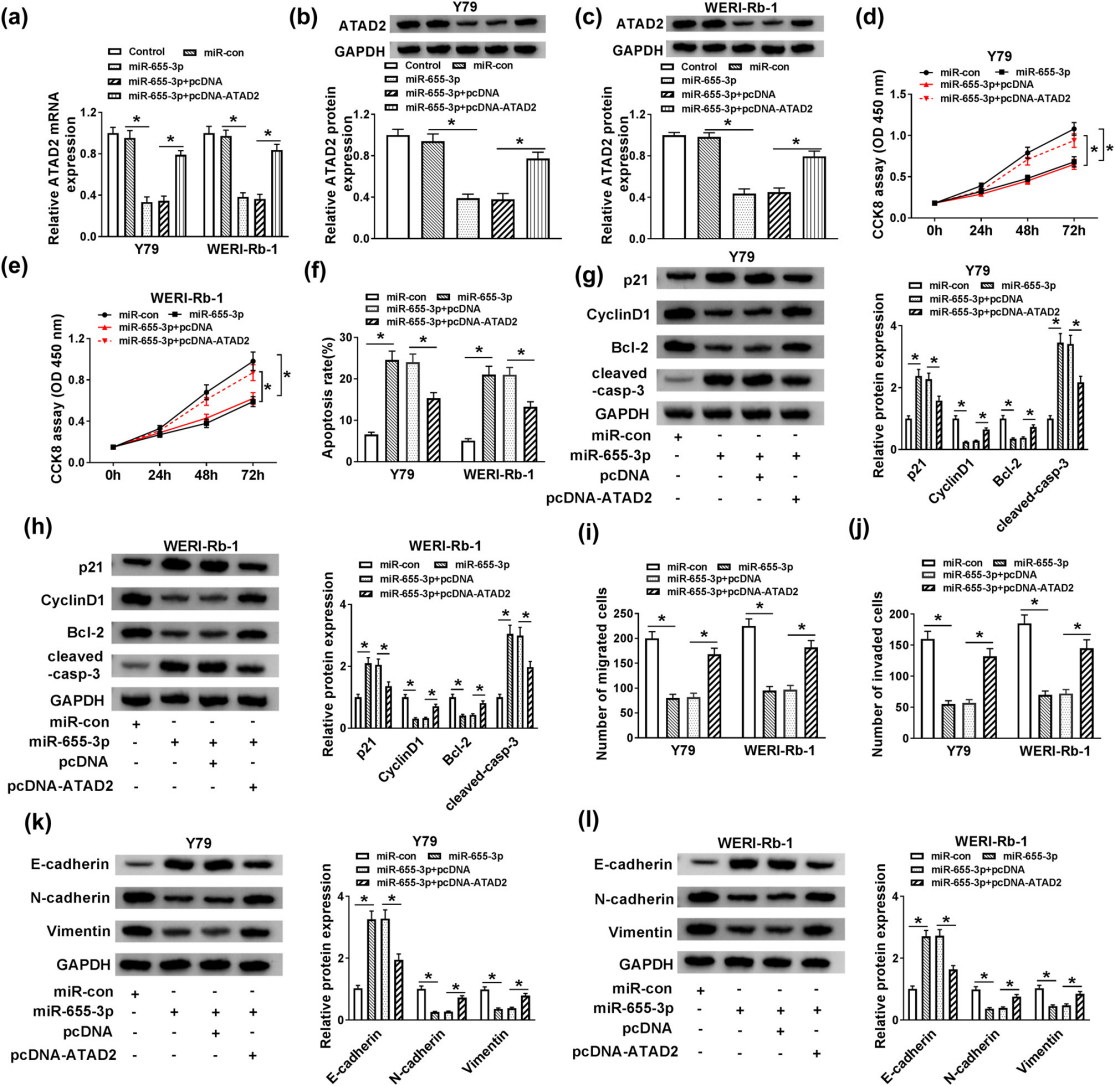
miR-655-3p negatively regulated ATAD2 expression to impede cell proliferation and impel apoptosis in Y79 and WERI-Rb-1 cells. (a–c) The Y79 and WERI-Rb-1 cells were transfected with Control, miR-con, miR-655-3p, miR-655-3p + pcDNA, or miR-655-3p + pcDNA-ATAD2. The levels of ATAD2 mRNA and protein were detected via RT-qPCR and western blot assay. (d–l) The Y79 and WERI-Rb-1 cells were transfected with miR-con, miR-655-3p, miR-655-3p + pcDNA, or miR-655-3p + pcDNA-ATAD2. (d and e) The cell viability was detected by CCK8 assay. (f) The apoptosis rate was measured through flow cytometry. (g and h) The protein levels of p21, Cyclin D1, Bcl-2, and cleaved-casp-3 were assessed via western blot assay. (i and j) The migration and invasion abilities were estimated by transwell assay. (k and l) The levels of E-cadherin, N-cadherin, and Vimentin protein were detected by western blot. *P < 0.05.

To investigate whether the effects of miR-655-3p on RB progression were mediated by ATAD2, cells were transfected with Control, miR-con, miR-655-3p, miR-655-3p + pcDNA, or miR-655-3p + pcDNA-ATAD2. As shown in Figure 6a–c, transfection of pcDNA-ATAD2 relieved the inhibition effect on ATAD2 mRNA and protein levels that were induced by miR-655-3p. Furthermore, the elevated ATAD2 partly reversed the repression impacts on cell viability, migration and invasion, and the promotion effect on cell apoptosis retarded by miR-655-3p in Y79 and WERI-Rb-1 cells (Figure 6d–f, i, and j). Finally, the transfection of pcDNA-ATAD2 mitigated the promotion effect on the protein levels of p21, cleaved-casp-3, E-cadherin as well as the restraint effect on the protein levels of Cyclin D1, Bcl-2, N-cadherin, and Vimentin in Y79 and WERI-Rb-1 cells caused by miR-655-3p mimics (Figure 6g, h, k, and l). These data implicated that miR-655-3p regulated cell proliferation, apoptosis, and migration and invasion in Y79 and WERI-Rb-1 cells by modulating ATAD2.

### 3.7 MALAT1 depletion impeded xenograft tumor growth *in vivo*

**Figure 7:**
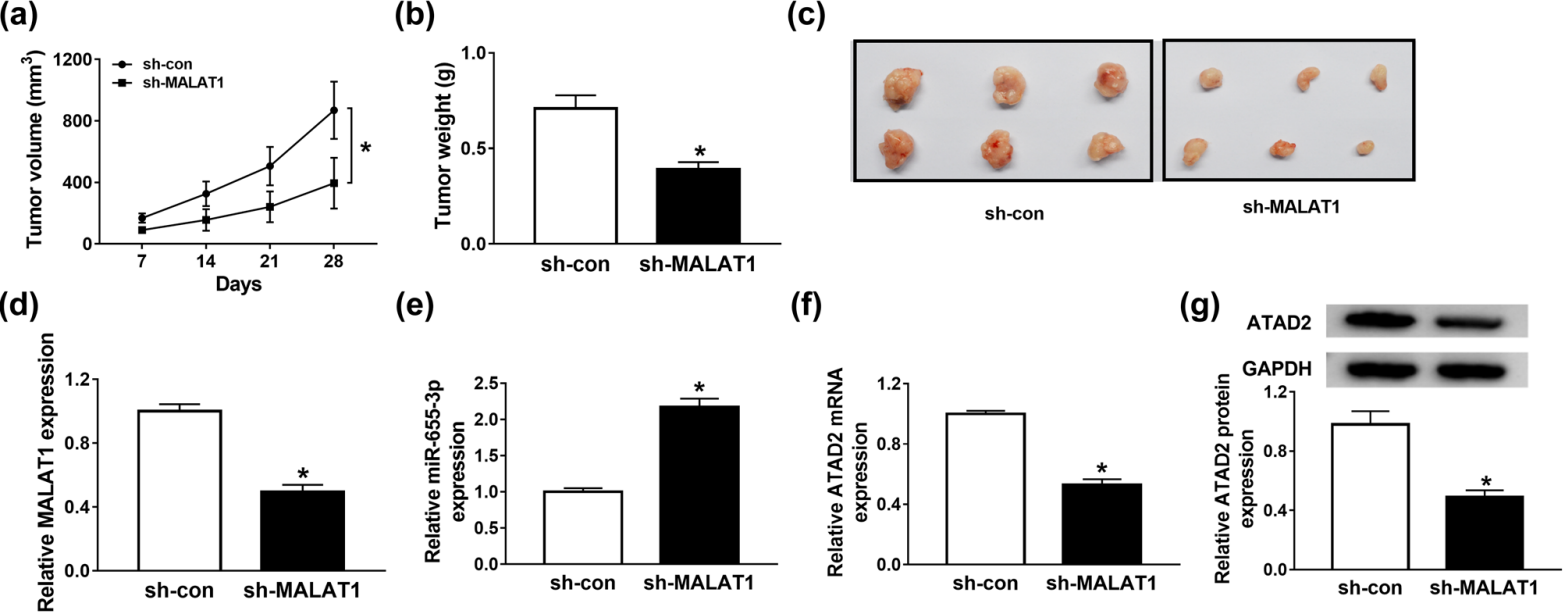
MALAT1 depletion impeded xenograft tumor growth *in vivo*. (a–g) The nude mice were injected with stable Y79 cell line expressing shRNA against MALAT1 or negative control. (a) The tumor volume was examined every 7 days for 28 days. (b) 28 days post-injection, the mice were executed, and tumors were removed and weighed. (c) Images of mice with subcutaneous transplanted tumors. (d–f) The expression levels of MALAT1, miR-655-3p, and ATAD2 in transplanted tumors were detected via RT-qPCR. (g) The protein level of ATAD2 in transplanted tumors was detected via western blot assay. *P < 0.05.

To verify the effects of MALAT1 *in vivo*, the nude mouse models of RB were established. The nude mice were injected with stable MALAT1 knockdown Y79 cells, and the tumor volume was measured every 7 days for 28 days. Twenty eight days upon injection, the mice were executed, the tumor tissues were removed, and stored in liquid nitrogen for further investigation. As shown in Figure 7a–c, MALAT1 depletion inhibited the tumor volume and weight in contrast with the sh-NC group. The expression of MALAT1, miR-655-3p, and ATAD2 in tumor tissue of nude mice were also analyzed. The levels of MALAT1, ATAD2 mRNA, and protein were decreased in the sh-MALAT1 group. However, the level of miR-655-3p was significantly elevated in the sh-MALAT1 group (Figure 7d–g). These results suggested that the silencing of MALAT1 inhibited xenograft tumor growth *in vivo*.

## 4 Discussion

RB is a rare type of intraocular cancer. At present, the early diagnosis and treatment of metastatic RB tumor are two critical problems for RB treatment. Convincing evidence indicated that MALAT1 was an oncogene in various types of cancer. In the current study, the roles of MALAT1 in RB were mainly explored. The results suggested that MALAT1 promoted RB progression partially through miR-655-3p/ATAD2 axis.

Emerging evidence demonstrated that the dysregulation of MALAT1 was implicated in tumor progression. For instance, a recent study disclosed that MALAT1 was strikingly elevated in ovarian cancer, and its depletion retarded cell growth and metastasis *in vitro* [9]. Another study in GC revealed that MALAT1 was conspicuously boosted in GC, and its depletion impeded cell growth, metastasis *in vitro* as well as induced apoptosis [10]. Similar results were reported in colorectal cancer [11], papillary thyroid cancer [12], and non-small cell lung cancer [13]. In this study, MALAT1 was highly expressed in RB tumors and cells. Meanwhile, MALAT knockdown blocked cell growth, metastasis, and EMT but enhanced apoptosis. In addition, MALAT1 depletion restrained xenograft tumor growth *in vivo*. These data were consistent with previous reports in RB [35,36], which demonstrated that MALAT1 played a vital role in RB progression.

miR-655-3p was encoded by 14q32 locus, which was reported to associate with the behaviors of tumor cells. For instance, Wang et al. reported that miR-655-3p was lowly expressed in non-small cell lung cancer and miR-655-3p mimics resulted in the impediment of cell metastasis [23]. Another study in hepatocellular carcinoma (HCC) demonstrated that the low expression of miR-655-3p in HCC promoted cell growth and metastasis [37]. In our research, miR-655-3p was downregulated in RB tumors and cells and exhibited a negative correlation with MALAT1. As lncRNAs exerted their biological function by exerting as ceRNAs for miRNAs, we speculated that miR-655-3p might be a target of MALAT1 in RB. As predicted, miR-655-3p contained the complementary binding sites of MALAT1, and it was further confirmed by dual-luciferase assay. Besides, miR-655-3p depletion could curb the effect of si-MALAT1 on cell growth, mobility, EMT, and apoptosis. These data implicated that MALAT accelerated RB progression by sponging miR-655-3p.

miRNAs could regulate the transcription and translation of their target mRNAs, thus affecting numerous biological processes [38]. To investigate the molecular mechanism of MALAT1/miR-655-3p involved in RB progression, we further predicted the target mRNA of miR-655-3p. ATAD2 was predicted as a target gene of miR-655-3p. ATAD2, located on human chromosome 8q24, is a member of the ATPase family containing an AAA domain which is vital for ATPase activity and protein assembly [39]. ATAD2 was reported to affect many biological processes in various types of tumors [40,41]. Accumulating evidence demonstrated that ATAD2 was involved in the progression of tumors. For example, a research in cervical cancer reported that the mRNA and protein levels of ATAD2 were elevated in cervical cancer, and the knockdown of ATAD2 impeded cell growth and metastasis [41]. Another study in HCC illustrated that ATAD2 was enhanced in HCC, and its knockdown regulated cell behaviors mediated by miR-372 [42]. In our research, ATAD2 was upregulated in RB tumors and cells and was negatively regulated by miR-655-3p. These results manifested that MALAT1 modulated ATAD2 expression to promote RB progression via miR-655-3p.

## 5 Conclusion

Taken together, we concluded that MALAT1 promoted ATAD2 expression to regulate cell proliferation, migration, invasion, apoptosis, and EMT in RB by sponging miR-655-3p. The new regulatory network sheds light on the mechanism of RB progression, which indicates that MALAT1 could be used as a diagnostic biomarker or a therapeutic target for RB. A recent research disclosed that combining noncoding RNAs with conventional cytotoxic chemotherapies for the treatment of cancers is a potential therapeutic strategy [19,43]. In follow-up study, we will focus on whether the MALAT1/miR-655-3p/ATAD2 axis affected the effectiveness of the chemotherapy for RB.
